# Omnipolar mapping technology used in a patient with Purkinje-related ventricular tachycardia

**DOI:** 10.1016/j.hrcr.2023.03.012

**Published:** 2023-03-24

**Authors:** Yousaku Okubo, Shogo Miyamoto, Naoto Oguri, Sho Okamura, Takehito Tokuyama, Yukiko Nakano

**Affiliations:** Department of Cardiovascular Medicine, Hiroshima University Graduate School of Biomedical and Health Sciences, Hiroshima, Japan

**Keywords:** Catheter ablation, Omnipolar mapping, Purkinje-related ventricular tachycardia, Ventricular tachycardia after myocardial infarction, High-density 3D mapping

**Key Teaching Points**

• Among Purkinje-related ventricular tachycardias (VTs) associated with ischemic heart disease, left posterior fascicular (LPF) VTs are characterized by relatively narrow QRS complexes, right bundle branch block morphology, and left axis deviation. Slowly conducting Purkinje fibers, similar to verapamil-sensitive VT, play a critical role in the VT circuit. However, the full extent of the re-entrant circuit has not been clarified.
• Novel omnipolar mapping technology using an HD grid catheter provided high resolution and precise mapping in a patient with Purkinje-related VT. Arrhythmias can be localized more accurately using the omnipolar mapping technology compared with the conventional bipolar mapping.
• Even with high-density mapping, the local activation time range included only about 50% of the tachycardia cycle length of the VT. In this case, the central isthmus of the VT (Purkinje-muscle junction) was located in the activation gap. Failure to record potentials via mapping on the endocardial side is most likely owing to location of the critical isthmus of the VT circuit (ie, Purkinje-muscle junction) in the deep myocardial layer.

## Introduction

Monomorphic sustained ventricular tachycardia (VT) in patients with ischemic heart disease is typically caused by re-entry circuits in the scarred area of the myocardium or the borders of the scar.^1^ However, Purkinje-related VTs, including re-entrant and focal VTs mediated by the His-Purkinje fiber network, also occur.^2^ Among Purkinje-related VTs, left posterior fascicular (LPF) VTs, which are characterized by relatively narrow QRS complexes, right bundle branch block morphology, and left axis deviation, also occur in the setting of structural heart disease.^3^ The re-entrant circuits of LPF-VT include the ventricular myocardium and part of the LPF. Hayashi and colleagues^4^ demonstrated that LPF-VTs after myocardial infarction (MI) can be successfully ablated at the site with diastolic Purkinje potential (P1). Although the slowly conducting Purkinje fibers, similar to verapamil-sensitive VT, play a critical role in the VT circuit, the full extent of the re-entrant circuit has not been clarified. We experienced a case of Purkinje-related VT in a patient with ischemic heart disease that was successfully treated by catheter ablation using omnipolar mapping technology, which provides clues for understanding the underlying mechanisms of this VT type.

## Case report

**Figure 1:**
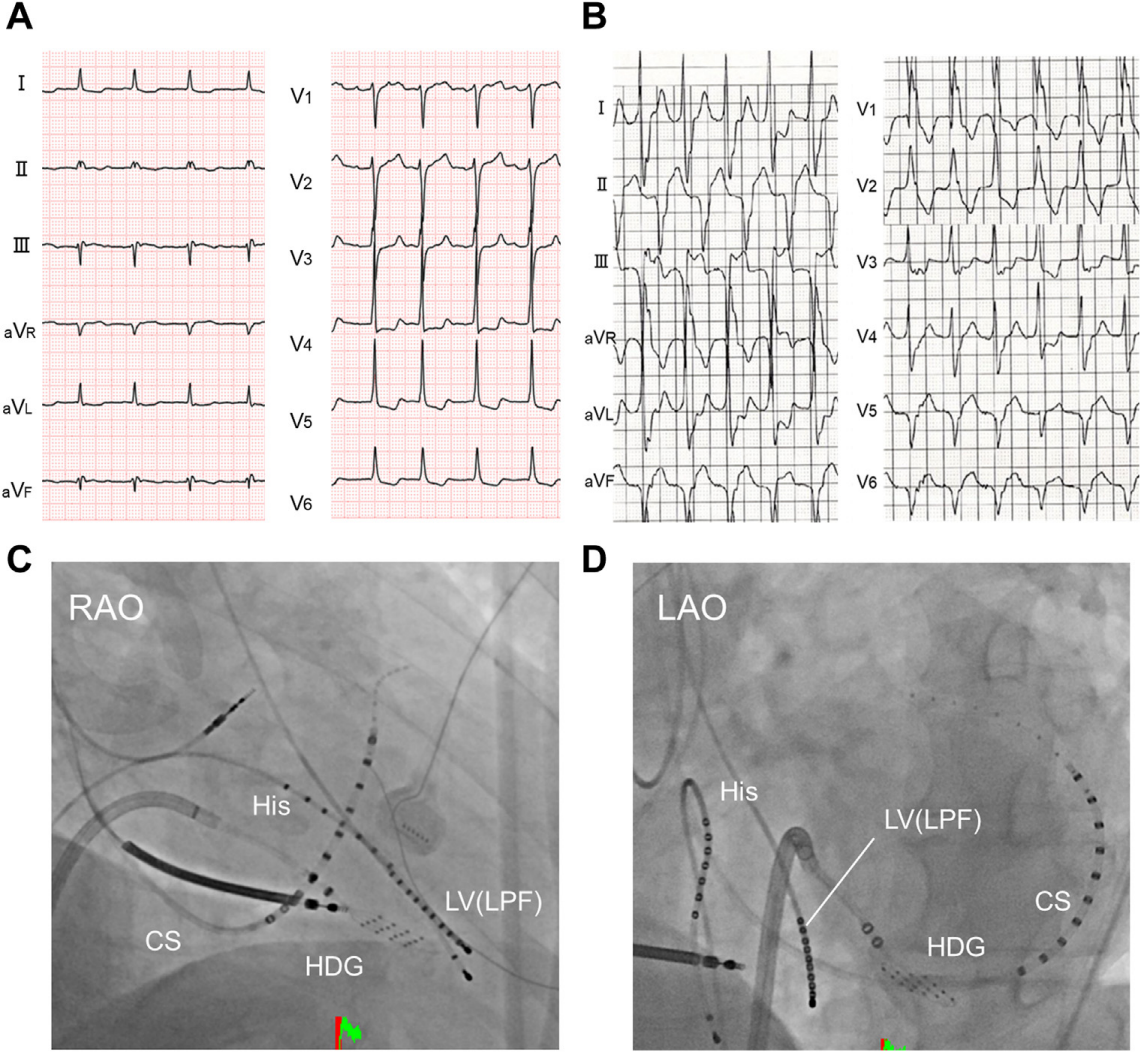
**A:** Twelve-lead electrocardiogram (ECG) during atrial fibrillation rhythm at baseline. **B:** ECG during monomorphic sustained ventricular tachycardia showed relatively narrow QRS complexes (134 ms), a right bundle branch block, and a superior-left axis deviation. **C, D:** Fluoroscopic images showing the catheter position. CS = coronary sinus; HDG = HD grid catheter; LAO = left anterior oblique; LPF = left posterior fascicle; LV = left ventricular; RAO = right anterior oblique.

A 56-year-old man with prior inferior wall MI and persistent atrial fibrillation (AF) was admitted to our hospital for VT recurrence. An implantable cardioverter-defibrillator was implanted and the patient was treated with oral antiarrhythmic drugs (amiodarone 200 mg/day and bisoprolol 5 mg/day). At baseline, 12-lead electrocardiography showed AF and abnormal Q waves in the inferior leads (Figure 1A). The electrocardiogram during monomorphic sustained VT revealed relatively narrow QRS complexes (134 ms), a right bundle branch block, and a superior left axis deviation (Figure 1)B. Cardiac echocardiography showed wall thinning and akinesia of the left ventricular (LV) inferior wall due to a prior MI. Because the monomorphic VT was resistant to antiarrhythmic drugs, catheter ablation was performed under deep sedation with intravenous dexmedetomidine.

**Figure 2:**
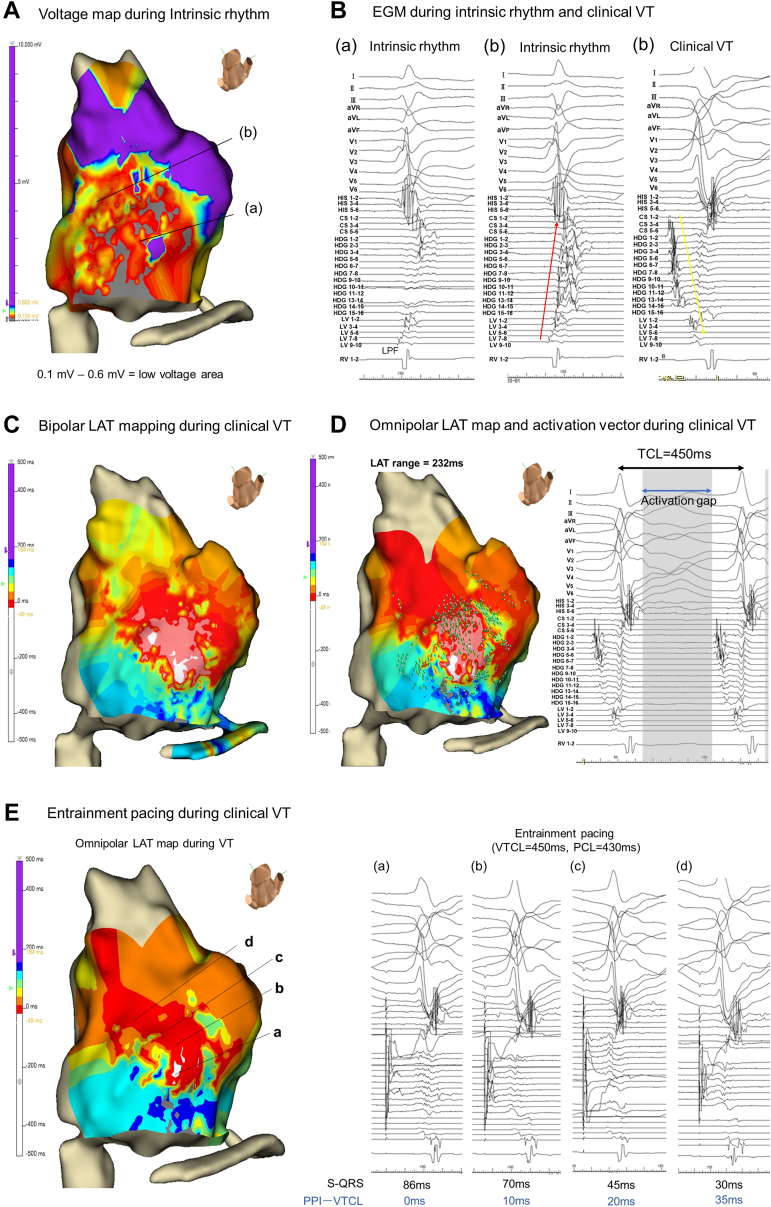
**A:** The omnipolar voltage map of the left ventricle shows the low-voltage area on the inferior wall. The low-voltage and dense scarred areas were defined as areas with peak-to-peak voltages of 0.1–0.6 and <0.1 mV, respectively. **B:** Endocardial electrograms (EGMs) during intrinsic atrial fibrillation (AF) rhythm and clinical ventricular tachycardia. (a) and (b) show the EGMs when the HD grid mapping catheter was located at (a) and (b) in panel (A), respectively. During intrinsic AF rhythm, the impulse propagated in the antegrade direction, from the proximal to distal sites in the left posterior fascicle (LPF) (indicated by the red arrow in Figure 2B). However, during clinical ventricular tachycardia (VT), the impulse propagated retrogradely from the distal to proximal sites of the LPF (indicated by the yellow arrow in Figure 2B). **C:** The bipolar local activation time (LAT) mapping during the clinical VT. **D:** Omnipolar LAT mapping and the activation vector. The activation vector revealed that the impulse propagated from the earliest activation site to the LPF and broke out from the LPF to the entire myocardium. The earliest LAT point was −48 ms and the latest LAT point was 184 ms, and the LAT range was only about 50% of the tachycardia cycle length (TCL). **E:** EGMs during entrainment pacing at each pacing site (a–d). The interval from the stimulus to the onset of the following QRS complex during entrainment (S-QRS) and the PPI after entrainment at each pacing site are shown on the bottom right side of (E).

Endocardial LV voltage mapping during intrinsic AF rhythm was performed using a duodecapolar liner electrode catheter (Inquiry™ Livewire™; Abbott, St. Paul, MN), a high-density mapping catheter (Advisor™ HD-Grid Mapping Catheter; Abbott, St. Paul, MN), and an EnSite™ X EP system (Abbott, St. Paul, MN). The duodecapolar liner electrode catheter was set where the left posterior fascicle potentials could be recorded (Figure 1C and 1D). The low-voltage and dense scar areas were defined as areas with peak-to-peak voltages of 0.1–0.6 and <0.1 mV, respectively. Mapping points were automatically acquired with the following settings: cycle length stability = 20 ms, catheter position stability = 1 mm/s, and point density = 1 mm. Low-voltage areas on the inferior wall were observed on the omnipolar voltage map of the LV (Figure 2A).

Endocardial electrograms (EGMs) during the intrinsic AF and the clinical VT are shown in Figure 2B. Panels (a) and (b) of Figure 2B show the EGMs when the HD grid mapping catheter was located at (a) and (b), respectively, in Figure 2A. During the intrinsic AF rhythm, the impulse propagated antegradely, from the proximal to the distal sites in the LPF (indicated by the red arrow in Figure 2B). However, during the clinical VT, the impulse propagated retrogradely, from the distal to the proximal sites of the LPF (indicated by the yellow arrow in Figure 2B).

The clinical VT induced by right ventricular (RV) burst pacing was hemodynamically stable (VT cycle length of 450 ms). Therefore, local activation time (LAT) mapping of the LV during the clinical VT was performed. Using conventional bipolar LAT mapping during the clinical VT, the earliest activation sites (EASs) were detected at 2 separate locations (Figure 2C). However, a single EAS location was identified using omnipolar mapping (Figure 2D). Furthermore, omnipolar LAT mapping and the activation vector revealed that the impulse propagated from the EAS to the LPF and the breakout occurred from the LPF to the entire myocardium. The Supplemental Video file shows propagation mapping during the clinical VT.

The earliest LAT point was −48 ms and the latest LAT point was 184 ms, and the LAT range was only about 50% of the tachycardia cycle length (TCL) (Figure 2D). We suspected that the VT was caused by a focal Purkinje VT rather than re-entry. Thus, entrainment pacing was performed from the RV apex. Constant fusion and progressive fusion were demonstrated with pacing from the RV apex at different rates (380 ms and 430 ms), suggesting a re-entrant mechanism. The postpacing interval (PPI) after entrainment pacing was 50 ms greater than the TCL of the VT.

We then performed entrainment pacing during the VT at the EAS, its surroundings, and the location where LPF potentials could be recorded. Figure 2E shows the 4 pacing sites and the EGM at each pacing site. The interval from the stimulus to the onset of the following QRS complex during entrainment (S-QRS) and the PPI after entrainment at each pacing site are shown in Figure 2E. The PPI minus the TCL was 0 ms and the S-QRS was 86 ms (less than 30% of the TCL) at the EAS (indicated by (a) in Figure 2E), suggesting that this site was the exit site of the VT circuit. Interestingly, entrainment pacing from a wide area where the Purkinje potential could be recorded resulted in concealed entrainment.

The difference between the PPI after VT entrainment from the RV apex and the TCL was longer than 30 ms; therefore, bundle branch re-entry VT was unlikely. Furthermore, the LPF, as the antegrade limb of the VT circuit, usually plays a role in bundle branch re-entry VT with right bundle branch block morphology. During the VT, the QRS morphology included a right bundle branch block and a superior left axis deviation. Thus, if the patient’s re-entrant mechanism was interfascicular re-entry, antegrade activation over the LPF would be observed. Therefore, we diagnosed a Purkinje-related VT mechanism as LPFVT.

**Figure 3:**
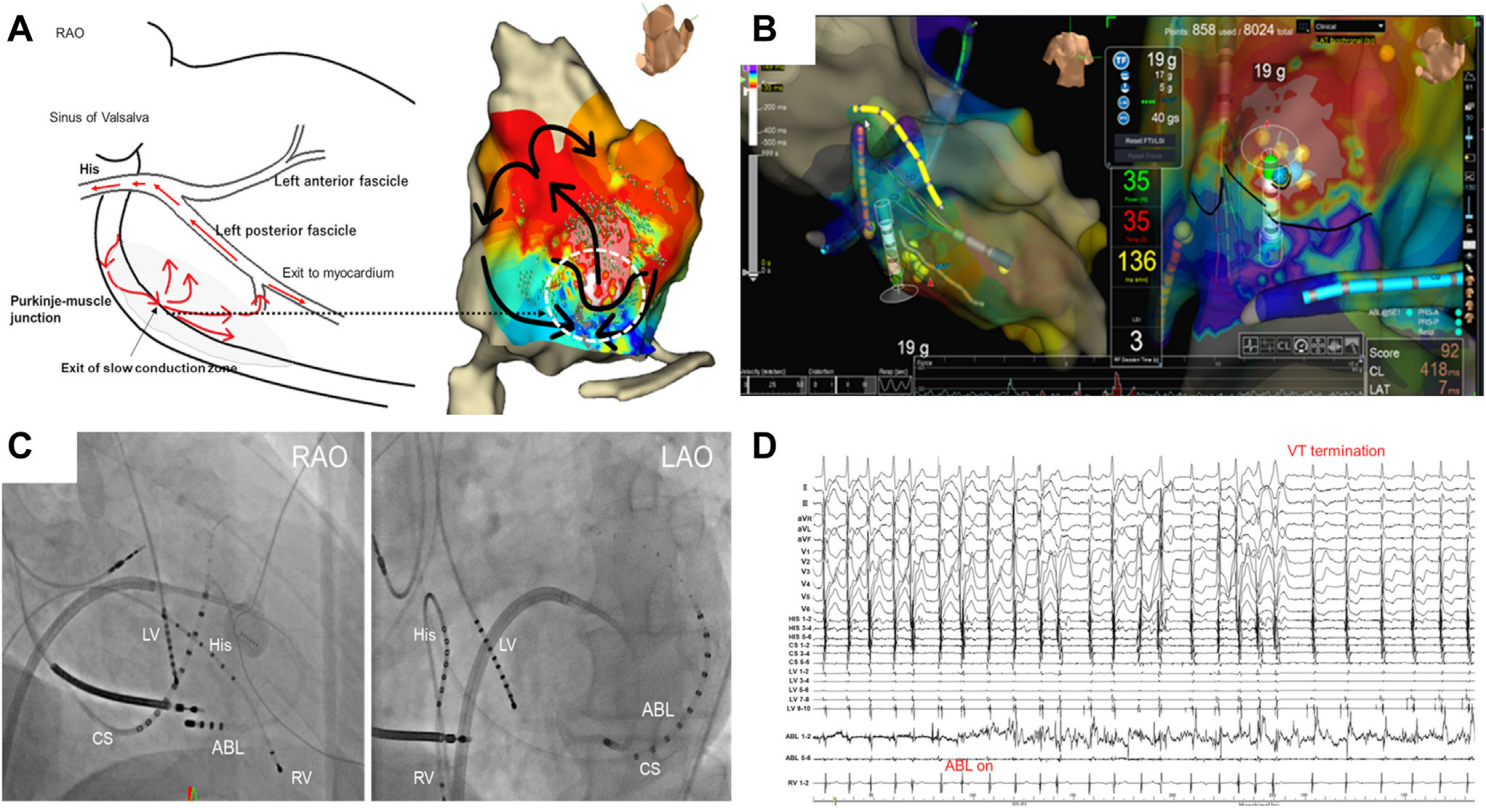
**A:** Schematic representation of the re-entrant mechanism for ventricular tachycardia (VT). **B:** Three-dimensional electroanatomic map during the radiofrequency application. An 8F, 4 mm flexible irrigated-tip catheter with 2-2-2-mm interelectrode spacing (TactiCathTM SE Ablation Catheter; Abbott, St. Paul, MN) was positioned at the early activation site during the VT. **C:** Fluoroscopic images showing the catheter position. **D:** The VT was immediately terminated when applying radiofrequency energy (35 W).

We positioned an 8F, 4 mm flexible irrigated-tip catheter with 2-2-2-mm interelectrode spacing (TactiCath™ SE Ablation Catheter; Abbott, St. Paul, MN) on the EAS. Application of radiofrequency energy (35 W) immediately terminated the VT (Figure 3B, 3C, and 3D). After the ablation, VT induction was attempted with programmed ventricular stimulation from the RV apex at 2 base cycle lengths (400 ms and 600 ms), with up to 3 extrastimuli. However, the clinical VT could not be induced. After discharge, the patient experienced no VT episodes during the 12-month follow-up period.

## Discussion

This is the first report of successful ablation for Purkinje-related VT using omnipolar mapping technology. Our major findings are 2-fold. First, the arrhythmia was localized using the novel omnipolar mapping technology and could be more accurately localized using the omnipolar mapping technology than via localization using the conventional bipolar mapping. The EAS was identified by the omnipolar mapping with an Advisor HD grid mapping catheter and an EnSite X EP System (Abbott). The EAS was the exit site of the VT isthmus, and the VT terminated immediately after the application of radiofrequency energy at this location.

The omnipolar mapping technology uses 3 unipolar signals and 2 orthogonal bipolar signals to analyze the voltage, activation direction, and wave speed independent of the catheter position. Furthermore, the omnipolar mapping technology avoids “bipolar-blindness,” a phenomenon in which activation wavefronts traveling perpendicular to the electrode pair are ignored during the electrophysiological mapping.^5^^,^^6^ With the improved mapping resolution, small potentials within the scar area owing to MI can be recorded with omnipolar mapping technology.

The HD grid catheter may also be suitable for recording Purkinje potentials. We reported the successful ablation of Purkinje-related VT using conventional bipolar mapping with the HD grid catheter.^7^ In this case, we were able to quickly, efficiently, and accurately map the diastolic Purkinje potentials using the omnipolar mapping technology.

The second major finding is that LAT mapping and the activation vector using the novel omnipolar mapping technology provide clues to help understand the mechanisms underlying this type of VT, although the full extent of the re-entrant circuit of the Purkinje-related VT remains unknown. An activation gap in the LAT mapping during the VT was observed between the exit site and the upstream of the circuit. The LAT mapping, the activation vector, and the results of the entrainment pacing indicated that the central isthmus of the VT was located in this activation gap area. Failure to record potentials via mapping on the endocardial side was most likely owing to the location of the critical isthmus of the VT circuit (ie, Purkinje-muscle junction) in the deep myocardial layer (Figure 3A). However, without reliable evidence, these views are only supposition. Hence, novel technology and a mapping device to record the deep layer of the myocardium are needed. To the best of our knowledge, this is the first case report of successful ablation for Purkinje-related VT using omnipolar mapping.

## Conclusion

A novel omnipolar mapping technology using an HD grid catheter provides good resolution and precise mapping in a patient with Purkinje-related VT.

## Appendix Supplementary Data

Supplemental Video A propagation mapping of the left ventricular during the clinical VT.
